# The Anti-Diabetic Effect of Non-Starch Polysaccharides Extracted from Wheat Beer on Diet/STZ-Induced Diabetic Mice

**DOI:** 10.3390/foods13172692

**Published:** 2024-08-26

**Authors:** Dounya Zad Oumeddour, Wen Lin, Chang Lian, Lei Zhao, Xinyi Wang, Liang Zhao, Liyun Guo

**Affiliations:** 1 Key Laboratory of Geriatric Nutrition and Health, Beijing Technology and Business University, Ministry of Education, Beijing 100048, China; douniazed.oumeddour@gmail.com (D.Z.O.); zhaolei@th.btbu.edu.cn (L.Z.); xaesi23@163.com (X.W.); 2 Beijing Engineering and Technology Research Center of Food Additives, Beijing Technology and Business University, Beijing 100048, China; 3 Beijing Key Laboratory of Beer Brewing Technology, Technical Center of Beijing Yanjing Brewery Co., Ltd., Beijing 101300, China; 13810081908@139.com (W.L.); 15712828393@163.com (C.L.)

**Keywords:** diabetes, dietary polysaccharide, non-starch polysaccharide, insulin resistance, high-sugar diet

## Abstract

Diabetes mellitus (DM), a major cause of mortality, is characterized by insulin resistance and β-cell dysfunction. The increasing prevalence of DM is linked to lifestyle changes and there is a need for alternative approaches to conventional oral hypoglycemic agents. Polysaccharides, particularly non-starch polysaccharides (NSPs), have been identified as promising hypoglycemic agents. Cereals, especially wheat, are key sources of dietary polysaccharides, with NSPs derived from wheat beer attracting significant interest. This study aimed to investigate the hypoglycemic and hypolipidemic effects of NSPs extracted from wheat beer in STZ-induced diabetic C57BL/6J male mice. The results showed that NSPs extract positively influenced blood glucose regulation, lipid profiles, and liver and kidney functions, by attenuating liver AST and kidney CRE levels in a dose-dependent manner. The NSPs demonstrated anti-oxidative and anti-inflammatory properties, potentially providing significant benefits in managing diabetes and its complications. Moreover, the study revealed the histoprotective effects of NSPs on the liver and pancreas, reducing lipid deposition, necrosis, and inflammation. These findings highlight the multifaceted advantages of NSPs and suggest their potential as effective agents in diabetes management. This study supports the need for further research into the therapeutic potential of NSPs and their application in developing innovative treatments for diabetes and its associated complications.

## 1. Introduction

Diabetes mellitus (DM), characterized by insulin resistance and β-cell dysfunction, manifests as a consequence of disrupted blood glucose homeostasis [1]. Diabetes stands prominently in the global health landscape, often securing the position of the fifth leading cause of mortality in high-income nations. By 2021, approximately 537 million adults worldwide were living with diabetes, resulting in around 6.7 million deaths annually, or one death every 5 s [2]. The rising global incidence of diabetes is believed to be linked to shifts in lifestyle and dietary patterns, particularly the increased consumption of rapidly digestible carbohydrates. Additionally, nourishment during pregnancy plays a crucial role in fetal programming, significantly impacting the child’s long-term health outcomes. These factors combined contribute to the growing prevalence of diabetes and its associated complications. Obesity is also believed to be closely linked to diabetes. Elevated levels of saturated fatty acids, particularly palmitic acid (C16:0) and stearic acid (C18:0), found during obesity, are highly associated with impaired insulin secretion and glucose intolerance. These fatty acids adversely affect β-cell function, a phenomenon known as glucolipotoxicity [3]. DM is linked to elevated risks of nephropathy, neuropathy, cardiovascular diseases, as well as retinopathy [4]. Oral hypoglycemic agents like biguanides, thiazolidinediones, and sulfonylureas effectively control hyperglycemia. However, they come with notable side effects, including hypoglycemia and gastrointestinal issues [5]. Hence, there is a pressing need for viable alternatives to mitigate diabetes complications while minimizing side effects. In recent years, there has been significant interest in exploring alternative medications to address diabetes. Polysaccharides, among dietary bioactive compounds, have been identified as promising hypoglycemic agents [6,7,8]. Plant non-starch polysaccharides (NSPs) have been shown to enhance insulin levels and promote hepatic glycogen accumulation, leading to a reduction in blood glucose levels in streptozotocin (STZ)-induced diabetic rats [9]. Additionally, they maintained islet structure by inhibiting β-cell apoptosis [10] and increased the cells’ insulin content [11]. NSPs have also been shown to downregulate diabetes-induced pro-inflammatory cytokines secretion in diabetic mice [12]. Moreover, they prevent liver fibrosis by inhibiting liver lipid synthesis [13] and reduce serum hepatic markers such as aspartate transaminase (AST) and alanine transaminase (ALT) [14]. NSPs can also inhibit the development of type 2 diabetes mellitus (T2DM) by decreasing oxidative stress, as evidenced by decreased levels of malondialdehyde (MDA) [15].

Dietary polysaccharides can be categorized into starch and NSPs, also known as dietary fiber. NSPs are a complex of numerous monosaccharide units, distinct from starch, linked together through glycosidic bonds. The primary constituents of NSPs are the polysaccharides found in plant cell walls, including cellulose, hemicellulose, and pectin. Additionally, NSPs encompass plant gums, mucilages, and hydrocolloids [16] and usually represent less than 10% of the grain’s weight. Cereals constitute the primary source of dietary polysaccharides for humans, contributing 50% of the total intake [17]. Wheat, among cereals, is particularly abundant in NSPs, predominantly located in the bran [18].

The creation of cost-effective and potent alternatives to oral hypoglycemic agents, ensuring efficient glycemic control, safeguarding the functionality of β-cells, exhibiting weight neutrality or facilitating weight loss, with reduced side effects, holds practical significance for both the pharmaceutical and nutrition industries. The present study aims to investigate the hypoglycemic and hypolipidemic effects of NSPs extracted from wheat beer at various concentrations both in vitro and in vivo. In vitro, the study will focus on evaluating the inhibitory effects of NSPs on α-amylase and pancreatic lipase enzymes, as well as assessing the fat-binding capacity. In vivo, the study will investigate the effects of NSPs on serum lipid, glucose, and insulin levels, as well as serum markers of liver and kidney functions, in diabetic C57BL/6J male mice. Additionally, the study aims to evaluate the potential antioxidant and anti-inflammatory properties of NSPs. Liver lipid accumulation will be assessed, supported by histopathological staining of liver and pancreas tissues to identify any associated morphological changes, inflammation, and pancreatic β-cell arrangement in the different experimental groups.

## 2. Material and Methods

### 2.1. Reagents and Kits

The following reagents were used: absolute ethanol (Beijing Yili Fine Chemicals Co., Ltd., Beijing, China), trifluoroacetic acid (TFA) (Sangon Bioengineering, Shanghai, Co., Ltd., Shanghai, China), monosaccharides standards (Borealis Biologicals, Vienna, Austria), α-amylase (porcine pancreas), pancreatic lipase (porcine pancreas), dinitrosalicylic acid and p-nitrophenyl butyrate (Shanghai Yuanye Biotechnology Co., Ltd., Shanghai, China), streptozotocin (STZ) (USP grade, Shanghai Biyuntian Biotechnology Co., Ltd., Shanghai, China). The assay kits for total cholesterol (TC), triglyceride (TG), high-density lipoprotein cholesterol (HDL-C), low-density lipoprotein cholesterol (LDL-C), alanine aminotransferase (ALT), aspartate aminotransferase (AST), creatinine (CRE), blood urea nitrogen (BUN), glycogen, and malondialdehyde (MDA) were all purchased from (Nanjing Jiancheng Institute of Bioengineering Co., Ltd., Nanjing, China). Enzyme-linked immunosorbent assay kits (ELISA) for mouse insulin (INS), mouse glucagon-like peptide 1 (GLP-1), mouse glucagon, mouse interleukin-1β (IL-1β), mouse interleukin-6 (IL-6), and mouse tumor necrosis factor α (TNF-α), were all purchased from (Wuhan Hualianke Biotechnology Co., Ltd., Wuhan, China).

### 2.2. Extraction of NSPs

This study used the method described by Song et al. [19], with slight modification. The beer sample was subjected to precipitation and separation in absolute ethanol (1:4 *v*/*v*) at 4 °C for 12 h. Subsequently, the mixture was centrifuged at 10,000× *g* for 10 min, and the supernatant was discarded. The resulting pellet was dissolved in 4.8 mL of sodium acetate buffer (100 mM, pH 5.0). A reaction with (0.1%, W/W) amyloglucosidase was conducted in a water bath at 50 °C for 30 min, followed by incubation with equal volumes of (10%, W/W) trichloroacetic acid solution for 12 h. The solution was then heated in a boiling water bath for 10 min and centrifuged at 10,000× *g* for 10 min. Absolute ethanol was added to the supernatant (1:2 *v*/*v*), and after standing for 30 min, the mixture was subjected to centrifugation at 10,000× *g* for 10 min. The resulting precipitate was washed twice with ethanol (95%, V/V), and NSPs were obtained through natural air-drying.

### 2.3. Characterization of NSPs

Sixteen monosaccharides standards (fucose, rhamnose, arabinose, galactose, glucose, xylose, mannose, fructose, ribose, galacturonic acid, glucuronide, aminogalactose hydrochloride, glucosamine hydrochloride, N-acetyl-D-glucosamine, guluronic acid, mannuronic acid) were weighed in precise amounts, and configured as a standardized concentration solution to be used as a mixed standard. According to the absolute quantification method, the mass of different monosaccharides was determined and the molar ratio was calculated based on the molar mass of monosaccharides. An amount of 5 mg non-starch polysaccharide extract was weighed in an ampoule, followed by the addition of 2 mL of 3M TFA for hydrolysis at 120℃ for 3 h. The acid hydrolysis solution was then accurately pipetted into a tube and dried with nitrogen. Subsequently, 5 mL of deionized water was added, and the mixture was well mixed and 100 µL of the solution was pipetted and mixed with 900 µL of deionized water. After centrifugation for 5 min at 12,000× *g*, the supernatant was taken for analysis by ion chromatography (IC).

The chromatographic conditions were as follows: column: Dionex CarbopacTM PA20 (3 × 150 mm); mobile phases: A: H_2_O; B: 15 mM NaOH; C: 15 mM NaOH, 100 mM NaAc; flow rate: 0.3 mL/min; injection volume: 25 µL; column temperature: 30 °C; detector: electrochemical detector

### 2.4. Enzymatic Assays

#### 2.4.1. α-Amylase Inhibition

The inhibiting capacity of NSPs of α-amylase was conducted according to the method used by Cardullo et al. [20], with slight modification. NSPs extract was prepared at concentrations of 0.1, 0.5, 1.0, 1.5, 2.0, 2.5, and 3.0 mg/mL. Then 0.5 mL of NSPs sample was mixed with 0.5 mL of α-amylase solution (10 U/mL, 0.1M PBS, pH 7.0) and incubated at 37 °C for 10 min. Subsequently, 0.5 mL of starch solution with a mass concentration of 0.1% was added, and the mixture was incubated at 37 °C for 20 min. Afterward, 100 μL of dinitrosalicylic acid was added, and the solution was boiled in a water bath for 5 min. Following cooling, the absorbance value was measured at 540 nm. The inhibition rate of α-amylase activity was calculated using Formula (1) as follows:

Inhibition rate = [1 − (A_a_ − A_b_)/(A_c_ − A_d_)] × 100% (1)

where A_a_ is the absorbance of mixture enzyme/substrate in the presence of tested compound; A_b_ is the absorbance of the sample background group (equal volume of enzyme solution is replaced by buffer); A_c_ is the absorbance of the control group (equal volume of the sample solution is replaced by buffer); and A_d_ is the absorbance of the control background group (equal volume sample and enzyme solution is replaced by buffer).

#### 2.4.2. Lipase Inhibition

The inhibiting capacity of NSPs of pancreatic lipase was conducted according to the method described by Franco et al. [21], with slight modification. A solution of pancreatic lipase at a concentration of 2.5 mg/mL in PBS (20 mM, pH 7.3) was prepared. It was then centrifuged at 10,000× *g* for 5 min, and 0.1 mL of the resulting supernatant was mixed with 0.3 mL of NSPs samples prepared at concentrations of 0.1, 0.5, 1.0, 1.5, 2.0, 2.5, and 3.0 mg/mL. The mixture was thoroughly mixed and incubated for 15 min at 37 °C. Then 0.5 mL of butyrate solution was added to the mixture, thoroughly mixed, and incubated at 37 °C for 15 min. The absorbance value was measured at 405 nm. The inhibition rate of lipase activity was calculated using Formula (2) as follows:

Inhibition rate = [1 − (A_a_ − A_b_)/(A_c_ − A_d_)] × 100% (2)

where A_a_ is the absorbance of mixture enzyme/substrate in the presence of tested compound; A_b_ is the absorbance of the sample background group (equal volume of enzyme solution is replaced by buffer); A_c_ is the absorbance of the control group (equal volume of the sample solution is replaced by buffer); and A_d_ is the absorbance of the control background group (equal volume sample and enzyme solution is replaced by buffer).

#### 2.4.3. Determination of Fat-Binding Capacity

The fat-binding capacity of NSPs was assessed using a method adapted from Sangnark et al. [22]. In the modified protocol, 2 mL of peanut oil was mixed with 40 mg of NSPs extract and stirred at 200 r/min for 2 h at 37 °C. The mixture was then centrifuged at 2500× *g* for 20 min, and the oil layer was carefully decanted. The remaining mixture was dried under vacuum at 40 °C. The amount of fat bound was quantified as mg of fat bound/10 mg of sample.

#### 2.4.4. Determination of Cholesterol-Binding Capacity Assay

The method described by Song et al. [19] was used. Fresh egg yolk was diluted with deionized water (1:5, V/V). Subsequently, a mixture of 4 mL of egg yolk and 40 mg of NSPs extract was mixed and stirred at 200 r/min for 2 h at 37 °C. Afterward, the mixture underwent centrifugation at 2500× *g* for 20 min. The cholesterol content in the resulting supernatant was determined using a free cholesterol content assay kit. The result was expressed as mg bound cholesterol/g sample.

### 2.5. Animal Experimental Design

Fifty male 8-week-old clean-grade C57BL/6J mice were housed in a clean and hygienic environment (room temperature 24 ± 2 °C, relative humidity 50% ± 5%, with a 12-h dark/light cycle) and had free access to food and water. After 1 week of adaptive feeding, nine mice were selected as the control group, and fed with a normal diet. Forty-one mice were fed an imported high-sugar and high-fat (HSHF) diet (66.5% basic diet, 10% lard, 20% sucrose, 2.5% cholesterol, 1% sodium cholate) as the experimental group. After 8 weeks, the mice in the experimental group were fasted for 12 h, and then injected STZ solution intraperitoneally at a dose of 50 mg/kg for 3 consecutive days (1% STZ in 0.1M citric acid–sodium citrate buffer, pH 4.2~4.5). The mice in the control group were fasted for 12 h and injected with the same dose of citric acid–sodium citrate buffer. One week later, the fasting blood glucose (FBG) of the mice was measured. Mice in the experimental group were considered successfully modeled for type II diabetes when their FBG levels exceeded 11.1 mmol/L twice consecutively.

The type II diabetic mice were then randomly divided into four groups (*n*  =  6): model group, low-dose group (L-NSPs, 100 mg/kg bw), medium-dose group (M-NSPs, 200 mg/kg bw), and high-dose group (H-NSPs, 400 mg/kg bw). NSPs were dissolved in ultrapure water and gavaged once daily. The control group and the model group were given the same dose of ultrapure water by gavage every day. During treatment, body weight and fasting blood glucose were measured once a week. An oral glucose tolerance test (OGTT) and insulin tolerance test (ITT) were performed at the end of the experiment. After 4 weeks of treatment, all mice were fasted for 12 h and whole blood samples were collected, serum was isolated by centrifugation, and aliquots were stored at −80 °C for later analysis. Following the sacrifice of the mice, the pancreas, liver, spleen, kidneys, gastrocnemius muscle, and adipose tissues (including epididymal, subcutaneous, perirenal, and subrenal fat) were immediately collected and weighed separately. A portion of the pancreas and liver was placed in 10% formalin solution for fixation and subsequent hematoxylin and eosin (H&E) staining. Part of the liver was frozen in liquid nitrogen before being transferred to −80 °C for oil red O staining, while the remaining liver was also cryopreserved at −80 °C for biomarker analysis. All animal experimental procedures in this research followed the National Institutes of Health’s guidelines for the treatment and use of laboratory animals. All animal procedures were conducted in accordance with the guidelines set by the Animal Ethics Committee of the Beijing Key Laboratory of Functional Food from Plant Resources (Permit Number: A330-2023-8, issued on 1 August 2023).

### 2.6. Oral Glucose Tolerance Test (OGTT) and Insulin Tolerance Test (ITT)

An OGTT was conducted at the end of the treatment. After 12 h of fasting, the OGTT was performed by giving glucose at 2 g/kg. Blood glucose was measured at 0, 30, 60, 90, and 120 min. An ITT was carried out by injecting all mice with insulin (0.75 U/kg) intraperitoneally after 4 h fasting. Orbital blood was taken before and after 30, 60, 90, and 120 min, and glucose level was measured. All experimental data were evaluated by calculating the area under the blood glucose curve (AUC).

### 2.7. Serum Biochemical Analysis

Fasting blood glucose (FBG) levels in the mice were systematically monitored on a weekly basis over a 4-week treatment period following a fasting duration of 12 h. TC, TG, HDL-C, LDL-C, AST, ALT, CRE, BUN, and MDA were measured using commercially available kits according to the manufacturer’s instructions. Insulin, glucagon, GLP-1, TNF-α, IL-6, and IL-1β were measured using ELISA kits according to the manufacturer’s instructions.

### 2.8. Determination of Liver Lipid and Glycogen Content

#### 2.8.1. Lipid Content in Liver Tissue

Liver TC and TG content was determined using commercially available kits according to the manufacturer’s instructions. Briefly, weighed liver tissue was homogenized with the lipid extracting solution provided in the kit, placed in an ice bath, and then centrifuged at 13,000× *g* for 10 min at 4 °C. Afterwards, the supernatant was used for measurement.

#### 2.8.2. Glycogen Content in Liver Tissue

Liver glycogen was determined using commercially available kits according to the manufacturer’s instructions. Briefly, weighed liver tissue was added to the extraction solution provided in the kit and placed in a boiling water bath for 20 min. Once all tissues were dissolved, the volume was adjusted to 5 mL with distilled water. The mixture was centrifuged at 8000× *g* at room temperature for 10 min and the supernatant was used for measurement.

### 2.9. Histological Analysis

Histological evaluation of liver and pancreas changes was conducted using H&E staining under a light microscope. Liver and pancreas tissues were fixed in 10% formalin at room temperature for 24 h. The fixed tissues were then embedded in paraffin and mounted on glass microscope slides. These sections were stained with hematoxylin and eosin (H&E) and examined under light microscopy at a magnification of 100×.

For the evaluation of fat deposition, liver sections with a thickness of 5–8 μm were embedded in OCT medium, mounted on glass slides, and stained with Oil Red O. These sections were examined at a magnification of 200×. The digital images were then analyzed using ImageJ (1.46r), where the lipid-positive areas (stained red) were identified and measured. The quantification was based on calculating the average optical density of the lipid-positive area ratios across multiple fields of view for each sample. These ratios were then used to determine the extent of lipid droplet deposition in the different experimental groups.

### 2.10. Statistical Analysis

SPSS 17.0 software was used to compare the means of all group pairs using one-way ANOVA, multivariate repeated measures ANOVA, and Tukey’s test for further analysis of the differences between the groups. All statistical tests were two-tailed, with *p* < 0.05 indicating a significant difference. GraphPad prism 8 software was used for plotting.

## 3. Results

### 3.1. Monosaccharides Composition of Extracted NSPs

**Figure 1:**
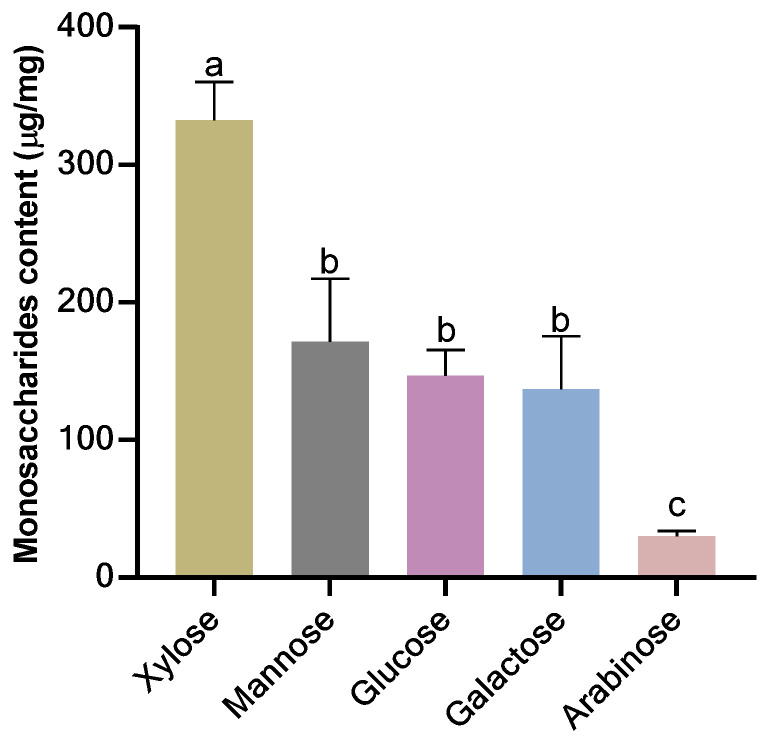
Monosaccharides composition of extracted NSPs. All data are shown as mean ± SD. Labeled means without a common letter differ significantly (*p* < 0.05).

NSPs were obtained using the anhydrous ethanol precipitation method, the purity of the extract was estimated at 89.96%, and the monosaccharides composition of NSPs was identified by comparing the retention time with standards. As shown in Figure 1, NSPs, a heteropolysaccharide, consisted of five monosaccharides including xylose, mannose, glucose, galactose, and arabinose at 332.4, 171.72, 146.64, 136.96, and 30.05 µg/g extract, respectively.

### 3.2. Enzyme Activity Inhibition Effect of NSPs

The inhibitory effects of NSPs extract at different concentrations on the activities of α-amylase and pancreatic lipase are summarized in Table 1.

As the concentration of NSPs increased, there was a corresponding rise in the inhibition rate of α-amylase activity. The most noticeable change in α-amylase inhibition occurred within the concentration range of 1.5 mg/mL to 2.0 mg/mL, with the inhibition rate increasing significantly from 43.18% ± 0.27% to 70.45% ± 0.52%. Beyond 2.0 mg/mL, the rate of inhibition slowed down, reaching a maximum of 81.82% ± 0.85%. Simultaneously, NSPs extract demonstrated an inhibitory effect on pancreatic lipase activity, with the inhibition rate exhibiting a positive correlation with sample concentration. The fastest increase in pancreatic lipase inhibition occurred between 1.5 mg/mL and 2.0 mg/mL, reaching a stabilization point and culminating in a 45.73% ± 1.01% inhibition rate at a concentration of 3.0 mg/mL.

### 3.3. Lipid-Binding Capacity of NSPs Extract

The fat-binding capacity test demonstrated that NSPs extract exhibited a binding ability of 5.92 mg/10 mg. Additionally, the cholesterol-binding test, employing the egg yolk method, revealed that NSPs displayed a binding capacity of 3.75 mg/g.

### 3.4. Effect of NSPs on Mice Body Weight and Organ Coefficient

**Figure 2:**
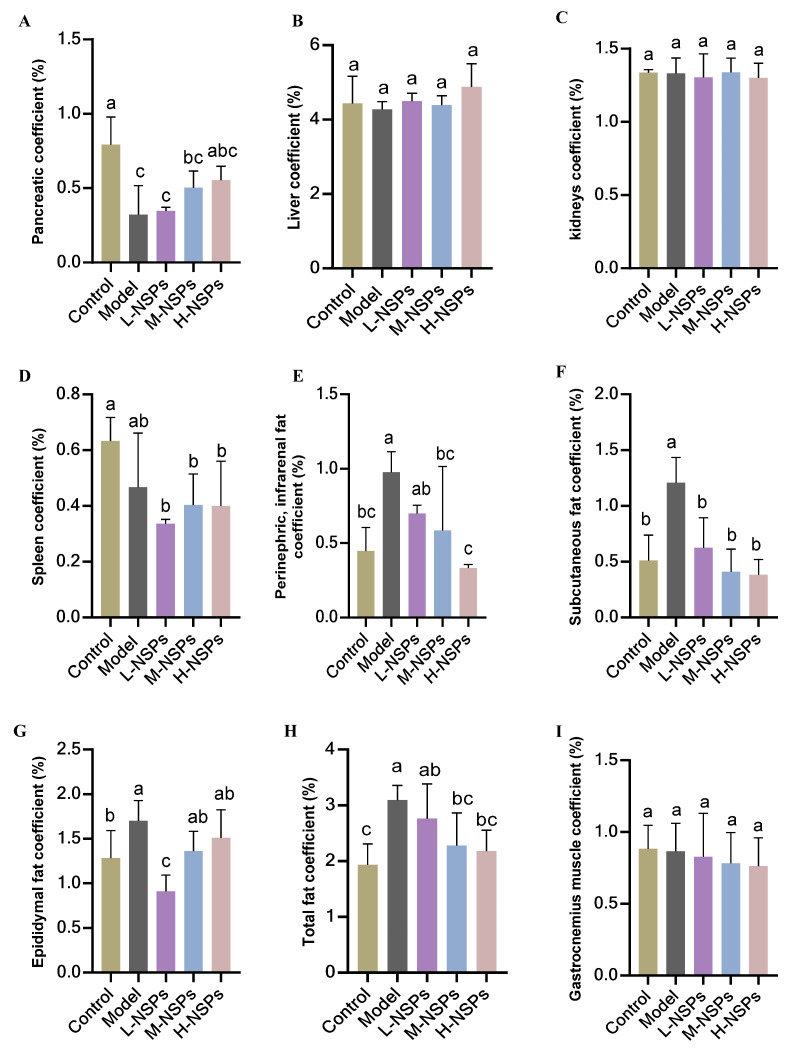
Organs, fat, and gastrocnemius muscle coefficient of the mice post NSPs treatment. The organ coefficient of pancreas (**A**), liver (**B**), kidneys (**C**), spleen (**D**), perirenal and infrarenal fat (**E**), subcutaneous fat (**F**), epididymal fat (**G**), total fat (**H**), and gastrocnemius muscle (**I**) are shown. All data are shown as mean ± SD. Labeled means without a common letter differ significantly (*p* < 0.05).

As shown in Table 2, after 4 weeks of treatment with NSPs, diabetic mice showed no significant difference in the final body weight in experimental groups compared to control and model groups (*p* > 0.05). No significant difference in food intake was recorded among groups (*p* > 0.05). The organ coefficient of the mice was calculated as the ratio of the weight of an organ to the body weight. In normal circumstances, the organ coefficient remains constant and organ weight changes are considered as a sensitive indicator of treatment-related effects [23]. If the organ index increases, it may indicate oedema or inflammation of the organ, but a drop in the index signals atrophy or damage to the organ. In the present study, NSPs-treated groups showed no significant difference (*p* > 0.05) in organ coefficients of liver, kidney, and gastrocnemius muscle compared with the control and model groups as illustrated in Figure 2. The analysis of spleen coefficients revealed no statistically significant differences (*p* > 0.05) between the different groups. Similarly, there were no significant differences (*p* > 0.05) observed between the model group and the various treated groups. However, the pancreatic coefficient exhibited a statistically significant decrease in both the model group (*p* < 0.001) and low-dose group (*p* < 0.001) when compared to the control group. Conversely, the medium-dose and high-dose groups demonstrated a significant elevation in pancreatic coefficient. Notably, the pancreatic coefficient in the high-dose group was comparable to that of the control group, with no statistically significant difference observed (*p* = 0.11). These results suggest that NSPs at a high dose of 400 mg/kg BW exhibited a positive effect on pancreas organ health. Perirenal, subrenal, and epididymal fat did not significantly differ (*p* > 0.05) across the groups, despite some observed variances. Compared with the model group, the subcutaneous fat coefficient of the control and treated groups at different doses was significantly lower (*p* < 0.05). Furthermore, there was a significant difference (*p* < 0.05) in the total fat coefficient between the model group and the middle-dose, high-dose, and control groups. Nonetheless, there was no discernible variation in the total fat coefficient between the middle-dose and high-dose groups and the control group (*p* > 0.05).

### 3.5. The Effect of NSPs on OGTT and ITT in the Diabetic Mice

**Figure 3:**
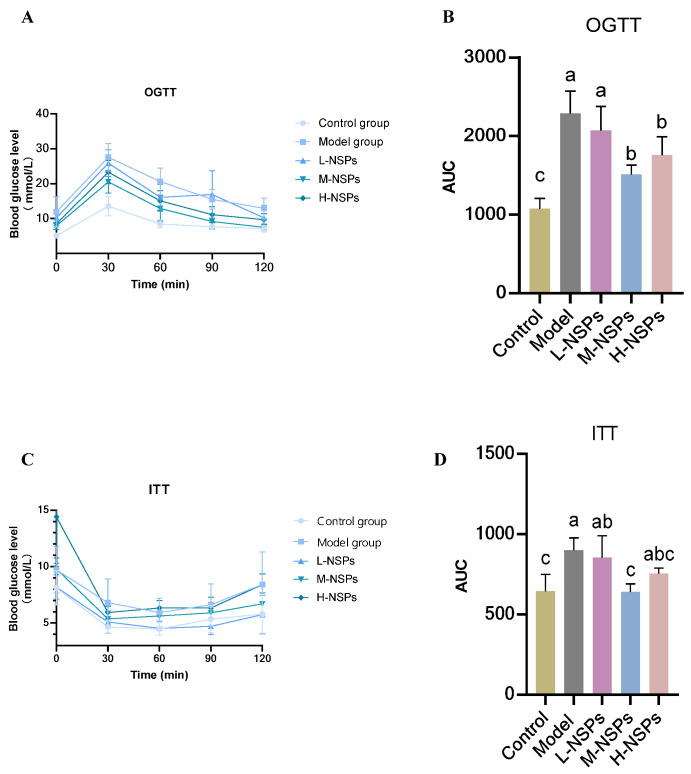
Glucose change in oral glucose tolerance test (OGTT) and insulin tolerance test (ITT). (**A**) OGTT curve, (**B**) histograms represent the area under the glucose curve in OGTT, (**C**) ITT curve, (**D**) histograms represent the area under the glucose curve in ITT. All data are shown as mean ± SD. Labeled means without a common letter differ significantly (*p* < 0.05).

The OGTT and ITT were conducted at the end of the treatment period, as illustrated in Figure 3. Following oral administration of 2 g/kg glucose to the mice, the blood glucose concentrations reached a peak within 30 min. Blood glucose concentrations and the corresponding AUC values, during OGTT and at all time points, were found to be significantly elevated in both the model group (*p* < 0.001) and low-dose group compared (*p* < 0.001) to the control group. Conversely, the middle-dose group and high-dose group exhibited decreased blood glucose levels and AUC at all time points, as shown in Figure 3A,B. The serum concentrations of glucose at 60–120 min were significantly decreased in the medium-dose group and high-dose group, reaching 7.5 ± 0.93 and 9.7 ± 1.73 mmol/L, respectively, compared to the model group which kept higher blood glucose concentration (12.9 ± 2.95 mmol/L). At the end of the experiment, the low-dose group also reached a significant decrease to 10.2 ± 3.57 mmol/L.

As shown in Figure 3C,D, the depiction of blood glucose levels following insulin treatment along with the area under the curve (AUC) of diabetic mice in both the model group and low-dose group exhibited a noteworthy elevation compared to the control group at all time points. Conversely, the middle-dose group and high-dose group demonstrated reduced blood glucose levels and AUC at all time points. Moreover, within the medium-dose group, the blood concentrations of glucose exhibited a significant decrease during the 60–120 min interval, attaining 5.74 ± 1.7, compared to the model group (*p* = 0.02) and low-dose group (*p* = 0.011) where the blood glucose level was reaching higher values (8.4 ± 0.96; 8.42 ± 2.87 mmol/L).

### 3.6. The Effect of NSPs on Mice Fasting Blood Glucose

**Figure 4:**
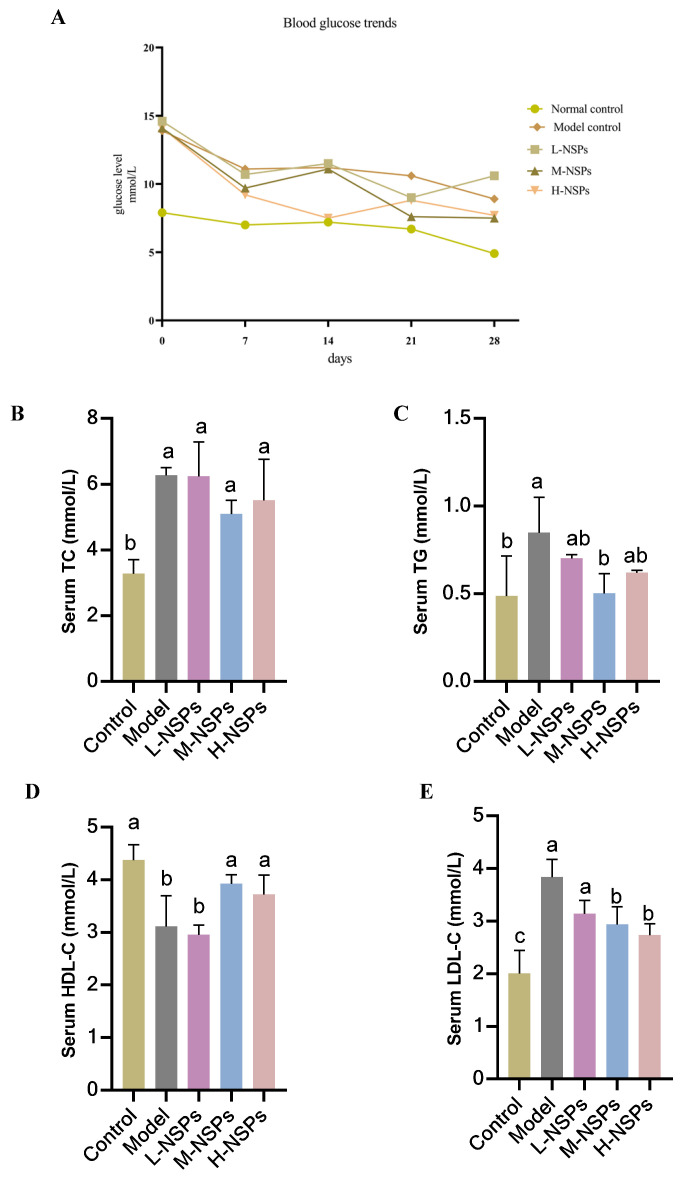
Fasting blood glucose (FBG) levels and serum lipid levels post NSPs treatment. (**A**) FBG levels, (**B**) total cholesterol (TC), (**C**) triglyceride (TG), (**D**) high-density lipoprotein cholesterol (HDL-C), and (**E**) low-density lipoprotein cholesterol (LDL-C) levels. All data are shown as mean ± SD. Labeled means without a common letter differ significantly (*p* < 0.05).

Throughout the 4-week intervention period, FBG levels within the control group remained consistently stable, maintaining values within the range indicative of control and low levels (8.9 ± 1.02–4.9 ± 1.05 mmol/L), as illustrated in Figure 4A. Nevertheless, FBG levels exhibited a gradual and notable increase in both the model group and treated groups, reaching values significantly higher than those observed in the control group (*p* < 0.05) during the first experimental week. After 2 weeks of treatment, the model group, low-dose group, and medium-dose group maintained elevated FBG levels (11.2 ± 0.8; 11.5 ± 1.2; 11.1 ± 1.04 mmol/L, respectively), in contrast to the high-dose group, which exhibited a notable decrease in FBG reaching 7.5 ± 0.93 mmol/L. By the third week, the high-dose group displayed a significant decrease in FBG compared to the model group, reaching 8.8 mmol/L (*p* = 0.001). At the end of the experiment, both the medium-dose and high-dose groups exhibited lower FBG levels compared to the low-dose group (7.5 ± 0.97; 7.7 ± 0.8; 10.6 ± 1.3 mmol/L, respectively), but still not significantly different from model mice group FBG level (*p* > 0.05).

### 3.7. The Effect of NSPs on Serum Lipid Profile

As shown in Figure 4B–E, the control group exhibited significantly elevated levels of TC, TG, and HDL-C, while displaying lower levels of LDL-C compared to the model group (*p* < 0.05). Following a 4-week intervention with NSPs at varying doses, the low-dose group demonstrated no significant differences in TG and HDL-C levels when compared to the model group (*p* > 0.05). TG levels reached significant low levels (0.5 ± 0.11 mmol/L) in the medium-dose group, distinguishing it from the other experimental groups. HDL-C exhibited a significant increase in both the medium-dose and high-dose groups (3.92 ± 0.17; 3.72 ± 0.36 mmol/L) compared to the model group and low-dose group (3.11 ± 0.58; 2.95 ± 0.18 mmol/L) (*p* < 0.05). Importantly, there was no significant difference observed between the control group, the medium-dose, and high-dose groups (*p* > 0.05). Regarding LDL-C levels, M-NSPs and H-NSPs groups exhibited significantly lower levels compared to the model group (*p* = 0.001; *p* = 0.003). Regarding TC levels, the NSPs treatment exhibited no significant differences among the experimental groups after 4 weeks of treatment, indicating no notable change compared to the model group (*p* > 0.05).

### 3.8. The Effect of NSPs on Hepatic Enzyme Levels

**Figure 5:**
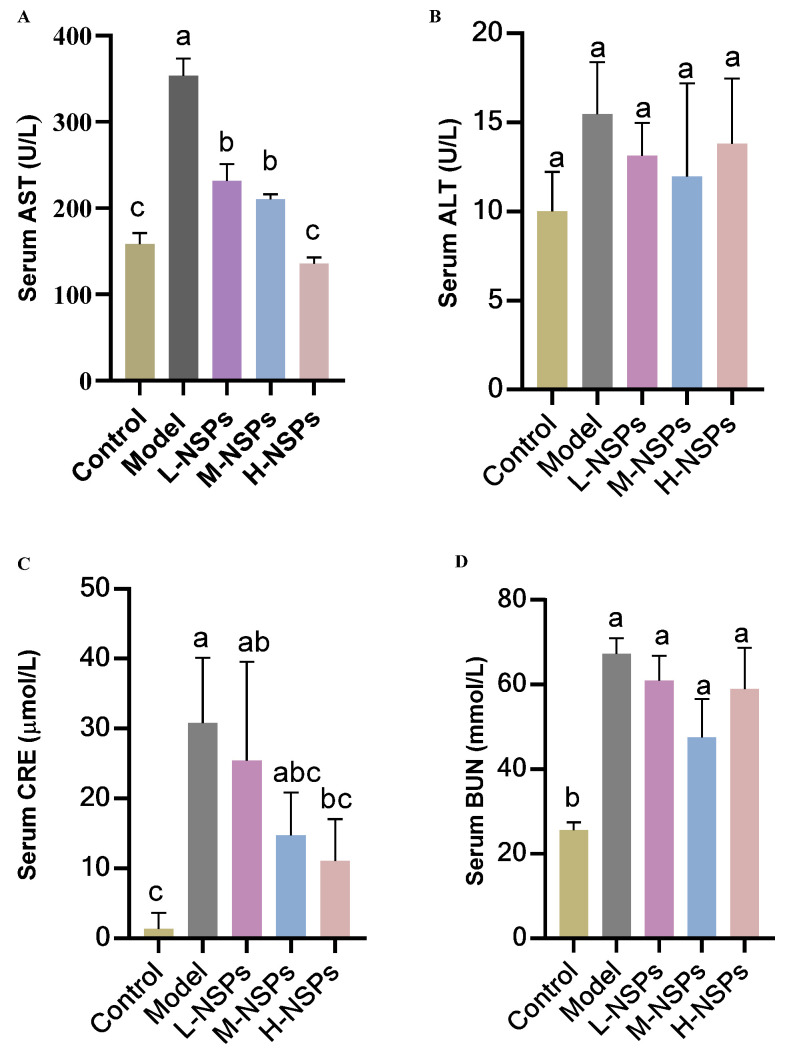
The effect of NSPs treatment on serum levels of aspartate aminotransferase (AST) (**A**), alanine aminotransferase (ALT) (**B**), creatinine (CRE) (**C**), and blood urea nitrogen (BUN) (**D**). All data are shown as mean ± SD. Labeled means without a common letter differ significantly (*p* < 0.05).

As depicted in Figure 5B, the results after 4 weeks of treatment revealed no substantial changes in ALT levels among the various groups of mice (*p* > 0.05). Conversely, there was a noteworthy increase in AST levels in the model group compared to the control group (*p* < 0.001) (Figure 5A). Notably, when comparing the model group to the different treated groups, it was observed that NSPs induced a significant decrease in AST levels in the low-dose group, medium-dose group, and high-dose group (231.52 ± 19.72; 210.03 ± 6.06; 281.91 ± 14.28 U/L vs. 353.64 ± 20.35 U/L, respectively).

### 3.9. The Effect of NSPs on Renal Enzyme Levels

As can be seen from Figure 5C, there was a notable and statistically significant increase in CRE levels within the diabetic model group when compared to the control group (*p* < 0.001). Intriguingly, after a 4-week treatment period with NSPs, a significant decrease (*p* < 0.05) in CRE levels was observed in treated mice as opposed to the model group. Notably, the high-dose group exhibited the most pronounced reduction in CRE levels (11.05 ± 6.02 µmol/L) among the treated groups. In terms of BUN serum levels, all diabetic mice, both in the model group and the treated groups, exhibited elevated levels of BUN compared to the control group (*p* < 0.05), with no significant differences observed among them (*p* > 0.05).

### 3.10. The Effect of NSPs on Serum Levels of Insulin, Glucagon, and Glucagon-like Peptide 1

**Figure 6:**
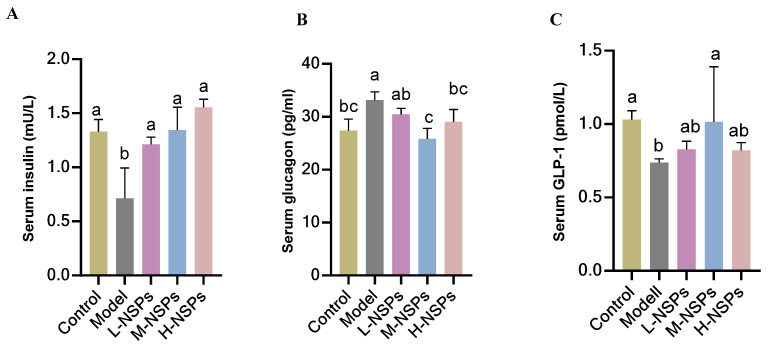
Serum insulin (**A**), glucagon (**B**), and glucagon-like peptide 1 (GLP-1) (**C**) levels after NSPs treatment. All data are shown as mean ± SD. Labeled means without a common letter differ significantly (*p* < 0.05).

As shown in Figure 6A, diabetic mice in the model group showed a significant decrease in serum insulin compared to the control group (0.71 ± 0.28; 1.33 ± 0.11 mU/L) (*p* = 0.01). However, treatment with NSPs demonstrated a significant increase in insulin levels compared to the model group (*p* < 0.05) across the low-dose, medium-dose, and high-dose treated groups (1.21 ± 0.06; 1.34 ± 0.21; 1.55 ± 0.07 mU/L, respectively). Notably, this increase in insulin levels brought the treated groups to levels comparable to those of healthy mice, with no significant differences observed (*p* > 0.05).

Conversely, glucagon serum levels exhibited a significant increase in the model group (*p* = 0.03) and low-dose group when compared to the control group. In contrast, the medium-dose group and high-dose group demonstrated an effective normalization of glucagon levels (25.80 ± 2.01; 29.05 ± 2.31 pg/mL, respectively), aligning them with the levels observed in the control group (27.42 ± 2.11 pg/mL), showing no significant difference (*p* > 0.05), as observed in Figure 6B. The GLP-1 results in Figure 6C, revealed that the medium dose of NSPs brought GLP-1 levels back to the control range comparable to the levels observed in the control group (*p* = 0.094). In contrast, GLP-1 levels in the low-dose and high-dose groups were close to those in the model group (*p* > 0.05).

### 3.11. The Effect of NSPs on Serum MDA Levels

**Figure 7:**
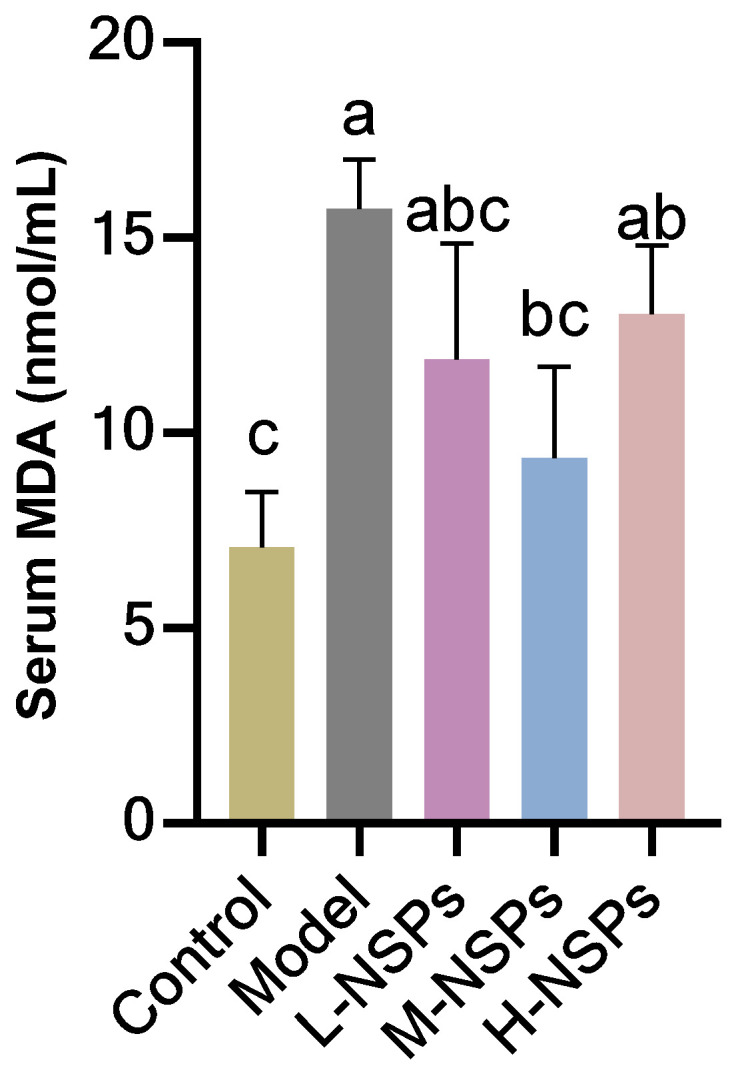
The effect of NSPs treatment on serum malondialdehyde (MDA) levels of diabetic mice. All data are shown as mean ± SD. Labeled means without a common letter differ significantly (*p* < 0.05).

The results shown in Figure 7 demonstrated a significant reduction in MDA levels with the administration of NSPs in the medium-dose group (18.75 ± 1.76 nmol/mL; *p* = 0.017) compared to the model group (15.83 ± 1.44 nmol/mL). Notably, the medium dose effectively normalized MDA levels, showing no significant difference from the levels observed in the control group (*p* = 0.65). While the high dose of NSPs also contributed to a decrease in MDA levels, this reduction did not reach statistical significance when compared to the model group (*p* = 0.059). This indicates that the NSPs treatment prevented substantial oxidative stress in diabetic mice.

### 3.12. The Effect of NSPs on the Inflammatory Response in Diabetic Mice

**Figure 8:**
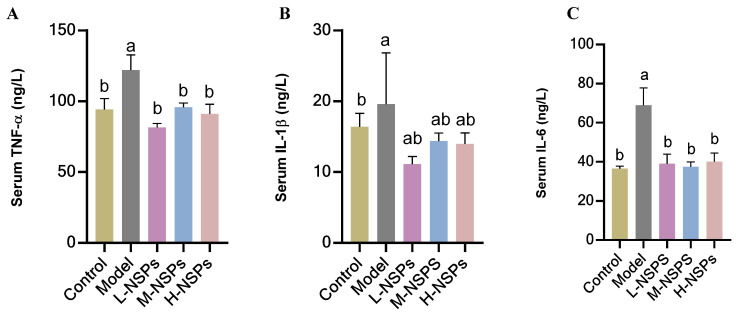
Serum TNF-α (**A**), interleukin-1β (IL-1β) (**B**), and interleukin-6 (IL-6) (**C**) levels of STZ-induced diabetic mice post NSPs treatment. All data are shown as mean ± SD. Labeled means without a common letter differ significantly (*p* < 0.05).

As can be seen from Figure 8, the serum levels of TNF-α and IL-6 exhibited a significant down-regulation (*p* < 0.05) after 4 weeks of NSPs treatment compared to the model group, effectively returning to normal levels comparable to non-diabetic mice. Conversely, IL-1β levels did not show a significant decrease when compared to the model group (*p* > 0.05).

### 3.13. The Effect of NSPs on Liver Lipid Accumulation of Diabetic Mice

**Figure 9:**
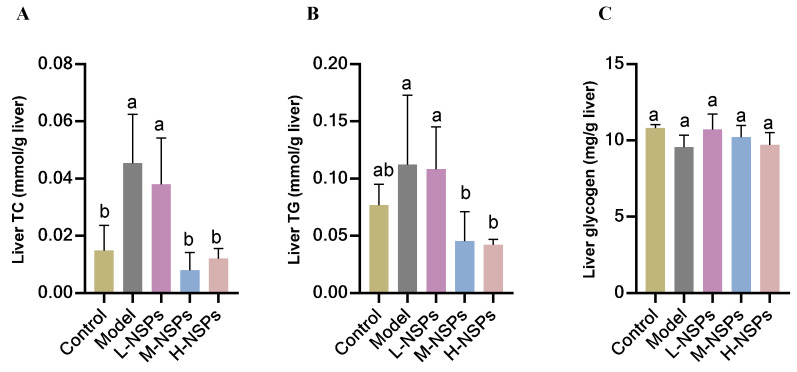
Liver total cholesterol (TC) (**A**), triglyceride (TG) (**B**), and glycogen (**C**) content of STZ-induced diabetic mice post NSPs treatment. All data are shown as mean ± SD. Labeled means without a common letter differ significantly (*p* < 0.05).

As illustrated in Figure 9A,B, the assessment of TC and TG levels in mice liver tissue highlighted that both the model group and the low-dose group displayed significantly elevated levels of TC (0.04 ± 0.01, *p* = 0.01; 0.03 ± 0.01, *p* = 0.058 mmol/g liver) and TG (0.11 ± 0.06, *p* = 0.047; 0.1 ± 0.03, *p* = 0.03 mmol/g liver) in comparison to the control group (TC 0.01 ± 0.008; TG 0.07 ± 0.01 mmol/g liver). Conversely, the medium-dose group and high-dose group exhibited a noteworthy and dose-dependent reduction in liver lipid content (TC: M-NSPs 0.008 ± 0.006, H-NSPs 0.01 ± 0.003 mmol/g liver; TG: M-NSPs 0.04 ± 0.02, H-NSPs 0.04 ± 0.004 mmol/g liver), ultimately reaching levels similar to those observed in the control group. This indicates that NSPs could improve lipid accumulation in the liver tissue, which can prevent future fatty liver diseases.

### 3.14. The Effect of NSPs on Liver Glycogen Content of Diabetic Mice

Glycogen levels were higher in the treated groups compared to the model group, although the difference was not statistically significant. Both treated and untreated groups maintained glycogen levels comparable to those of the control group, with no statistically significant differences observed (*p* > 0.05).

### 3.15. The Effect of NSPs on Liver Histology

#### 3.15.1. H&E Staining

**Figure 10:**
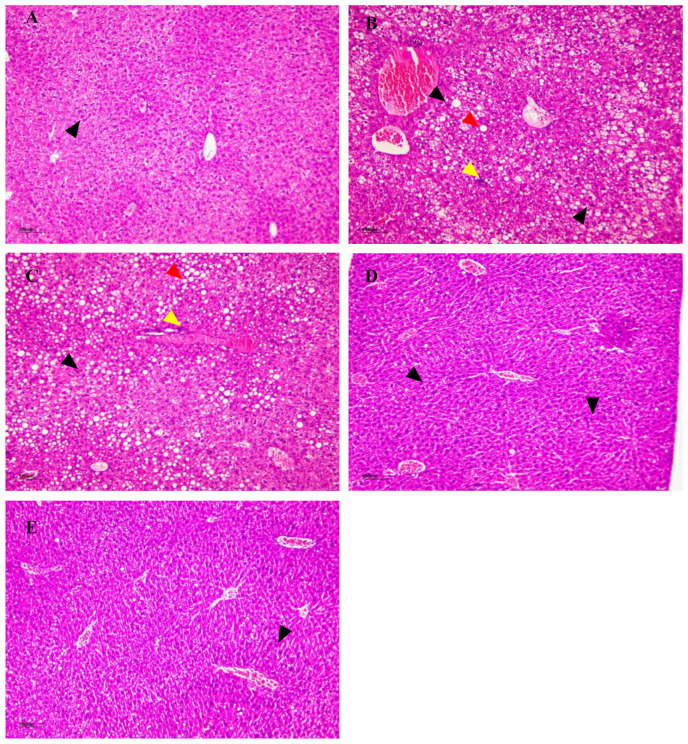
Hematoxylin and eosin (H&E) of liver tissue. (**A**) control group, (**B**) model group, (**C**) L-NSPs group, (**D**) M-NSPs group, (**E**) H-NSPs group. H&E magnification ×100; scale bar = 100 µm. Black arrow = hepatocytes; red arrow = fat deposition; yellow arrow = inflammatory cells.

H&E staining of liver sections from diabetic mice as shown in Figure 10, unveiled pronounced anomalies within the model group. Remarkably, hepatocytes exhibited disarrangement and hyperplasia, accompanied by the conspicuous presence of fat droplets. Further scrutiny disclosed hepatic lobular degeneration and necrosis, indicative of severe tissue damage. A marked increase in the inflammatory response contributed to a discernible presence of inflammatory cells. Additionally, hepatic sinusoidal congestion was evident, accentuating the perturbations in microcirculation within the liver. These findings collectively underscore the profound impact of diabetes on liver histology. Furthermore, in-depth analysis of the impact of different doses of NSPs revealed intriguing outcomes. The lowest dose of NSPs administered to treated mice did not confer protection against liver necrosis, inflammation, and the persistence of adipocytes already observed in the model group, as illustrated in Figure 10B,C. Contrastingly, the administration of medium and higher doses of NSPs effectively shielded the liver from the aforementioned abnormalities. This is evident in Figure 10D,E, where abundant, normally structured and arranged hepatocytes are observed. Notably, small amounts of adipocytes and mild inflammation are observed in discrete areas, reminiscent of the control group presented in Figure 10A. These findings underscore the potential dose-dependent protective effects of NSPs on liver histology in diabetes.

#### 3.15.2. Oil Red O Staining

**Figure 11:**
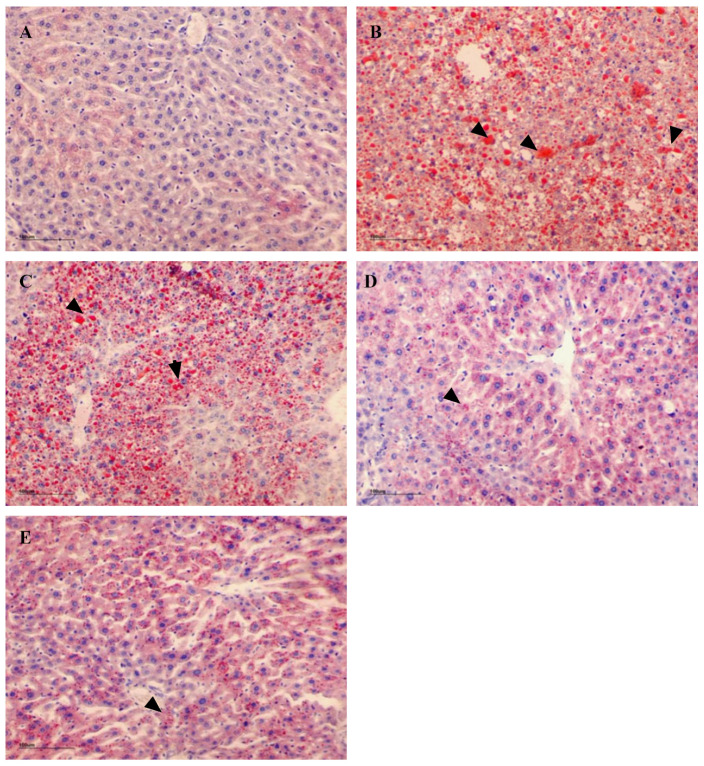
Oil Red O staining of liver tissue. (**A**) control group, (**B**) model group, (**C**) L-NSPs group, (**D**) M-NSPs group, (**E**) H-NSPs group. Lipid drops are judged by red staining. Magnification ×200; scale bar = 100 µm. Black arrow = fat deposition.

To confirm lipid deposition in the liver of the experimental mice, Oil Red O staining was employed to quantify fat droplets within hepatocytes (Figure 11). Histological analysis demonstrated a notable increase in intracellular lipid accumulation in the liver of the model group compared to the control group as illustrated in Figure 11A,B. This was accompanied by elevated hepatic TC and TG levels (TC: model 0.04 ± 0.01, control 0.01  ±  0.008; TG: model 0.11 ± 0.06, control 0.07 ± 0.01 mmol/g liver) along with a rise in the positive area ratio of optical density by 13.139% and 2.426%, respectively. Liver sections from the low-dose group exhibited fewer lipid droplets, as depicted in Figure 11C. However, there was no significant difference from the model group, as both groups displayed high levels of liver TG and TC (TG: L-NSPs 0.1 ± 0.03, model 0.11 ± 0.06; TC: L-NSPs 0.03 ± 0.01, model 0.04 ± 0.01 mmol/g liver). In contrast, liver sections from the medium-dose and high-dose groups revealed a significant reduction in lipid droplet deposition compared to the model group (Figure 11D,E), characterized by a notable decrease in liver TC and TG accumulation (TC: M-NSPs 0.008 ±  0.006, H-NSPs 0.01  ±  0.003; TG: M-NSPs 0.04 ± 0.02, H-NSPs 0.04  ±  0.004 mmol/g liver). The calculated ratio area was 3.71% for the medium-dose group and 2.862% for the high-dose group. These findings underscore the dose-dependent efficacy of the administered NSPs in mitigating hepatic lipid accumulation.

### 3.16. The Effect of NSPs on Pancreas Histology

**Figure 12:**
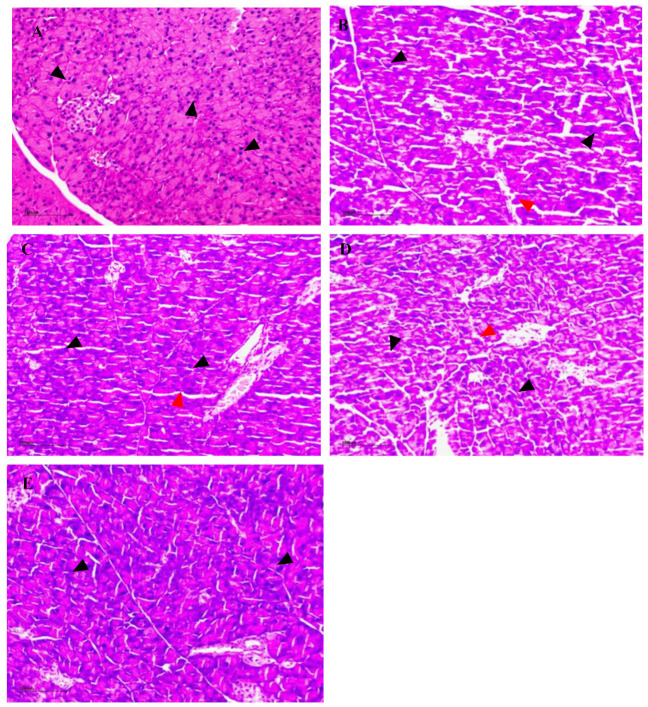
Hematoxylin and eosin (H&E) of pancreas tissue. (**A**) control group, (**B**) model group, (**C**) L-NSPs group, (**D**) M-NSPs group, (**E**) H-NSPs group. Magnification ×100; scale bar = 100 µm. Black arrow = islets of Langerhans; red arrow = interlobular space.

To evaluate the histologic alterations in the pancreatic tissues and the possible protective effect of NSPs on β-cell function in diabetic mice, H&E staining analysis was performed. A fully intact pancreatic islet structure in the control mice, characterized by the presence of regularly distributed and abundant pancreatic β-cells, and homogeneous arrangement with compact intercellular spaces in the pancreas, was observed as shown in Figure 12A. By contrast, the islets in diabetic model mice and treated mice at low NSPs concentration exhibited atrophy and severe damage, coupled with a conspicuous reduction in pancreatic β-cell distribution. Additionally, large inflammatory cell infiltrates were evident, highlighting the pronounced state of inflammation (Figure 12B,C). The pancreatic tissue structure in both the medium-dose and high-dose groups exhibited overall clarity. The acinar lobules remained predominantly intact, and the interlobular space appeared essentially normal. However, there were discernible instances of inflammatory cell infiltration, accompanied by a minor presence of abnormal pancreatic islet cells (Figure 12D,E). The results suggested that NSPs effectively improved the damaged islet structure in diabetic mice.

## 4. Discussion

DM, as previously mentioned, is a chronic condition characterized by impaired blood glucose regulation. It manifests through hyperglycaemia resulting from either target tissue insulin resistance, insufficient insulin production, or a combination of both [24]. Continuously elevated levels of blood sugar and lipids stand as primary contributors to the deterioration of organ functions in vital human organs, including the liver, eyes, and kidneys [25]. Additionally, dietary polysaccharides from various sources, each with distinct monosaccharide compositions, have been approved for their hypoglycemic properties, underscoring their potential in managing diabetes and alleviating its complications [26,27,28]. According to the chromatographic analysis of extracted NSPs, xylose and mannose were the main monosaccharides present in the extract. This differs from the monosaccharides composition of another beer sample, Song et al. [19], which demonstrated a distinct monosaccharides composition, where xylose and arabinose were the main constituents. This divergence in composition suggests potential influences from varying malt varieties and formulations utilized in the brewing process, highlighting the intricate impact of raw materials on the final product.

The gradual breakdown of starch into monosaccharides is facilitated by α-amylase, promoting the digestion and absorption of starch, and consequently resulting in elevated blood glucose levels [29]. Polysaccharides can bind to the active center of α-amylase, inhibiting its activity and reducing starch digestion, thereby contributing to a decrease in blood glucose levels [30]. Pancreatic lipase promotes the absorption of triglycerides in the body, and by inhibiting the activity of pancreatic lipase, it can reduce the absorption of lipid substances and lower blood lipid levels [31]. The activity of α-amylase in digesting carbohydrates contributes to elevated postprandial glucose levels in individuals with diabetes [32]. On the other hand, pancreatic lipase is the main factor responsible for dietary fat breakdown into smaller molecules of glycerol and fatty acids, facilitating their absorption by the body and integration into metabolic processes [33]. By inhibiting the function of these two enzymes, it is possible to effectively manage and control obesity, as well as postprandial hyperglycaemia, thereby mitigating the risk of diabetes development. In the present study, the findings revealed significant α-amylase and lipase inhibitory activity of the NSPs extract in a dose-dependent manner. The α-amylase inhibition reached a rate of 81.82% at a concentration of 3 mg/mL of the extract. This contrasts with the results reported by Song et al. [19], where the NSPs extract from a different beer brand showed no inhibitory activity on α-amylase. Additionally, inferior lipase inhibition activity was observed, with the highest concentration of 1.25 mg/mL demonstrating an inhibitory rate comparable to the lowest concentration of 0.1 mg/mL in the present extract, as previously detailed in Table 1. Dietary polysaccharides from *C. tinctoria*, composed mainly of galactose and arabinose, exhibited higher α-amylase inhibition rate of 59.24% at 1 mg/mL, compared to 27.27% in the present extract [34]. Conversely, a noteworthy observation is that heightened α-amylase inhibition activity is consistently linked with a range of adverse effects, including flatulence, diarrhea, bloating, and abdominal discomfort [35]. In the realm of scientific inquiry, it is increasingly recognized that a lower inhibitory of α-amylase activity may be considered more favorable due to its potential to mitigate these undesirable consequences. These findings underscore the crucial role of diverse monosaccharides composition in shaping the inhibitory activity of dietary polysaccharides. Dietary fiber extracted from *Kappaphycus alvarezii* and *Kappaphycus striatus* seaweed demonstrated lower inhibitory effect on pancreatic lipase activity compared to the present beer NSPs extract. At a concentration of 3.8 mg/mL, the seaweed extracts showed inhibition rates of 36% and 43%, respectively, compared to 45.73% at 3 mg/mL of NSPs extract [36].

Furthermore, it is noteworthy that compositional variations of NSPs also exert a significant impact on their effectiveness as hypolipidemic and hypoglycaemic agents. Assuming that NSPs remains unhydrated during their transit in the intestine by forming complexes with lipids in the digestive tract, this leads to the formation of large molecules. Consequently, this process efficiently impedes their absorption by the body, thereby preventing their entry into the systemic circulation [37] and may contribute significantly to their hypolipidemic effect [38,39]. To delve deeper into the hypolipidemic effects of the extracted NSPs, a comprehensive assessment was conducted through a lipid-binding capacity test. The results showed higher fat- and cholesterol-binding capacity (5.92 mg/10 mg and 3.75 mg/g, respectively) compared with the previously mentioned results reported by Song et al. [19], where NSPs samples showed a binding capacity of 3.30 mg/10 mg and 1.41 mg/mL, respectively. In contrast, NSPs from *Passiflora edulis* peel showed better fat-binding capacity of 10.38 g/g and a cholesterol-binding rate of 78.69% [40].

We further evaluated the effect of NSPs, at three different doses, on STZ-induced diabetic mice over a period of 4 weeks. The results indicated that both the model mice and those treated with various NSPs doses exhibited no significant differences in average body weight compared to the control group mice at the experiment’s conclusion, along with comparable food intake. However, the organ index results revealed that diabetic mice and those treated with a low dose of NSPs had a lower pancreas index, signifying a smaller pancreas size. The pancreas, a vital player in glucose metabolism through the regulation of blood glucose via insulin secretion, may undergo damage during STZ induction, leading to disturbances in insulin secretion [41]. This aligns with a commonly observed phenomenon associated with exocrine pancreatopathy in diabetes [42]. Notably, the medium and high doses of NSPs demonstrated an effective dose-dependent improvement in pancreatic organ size. This was consistent with previous results, suggesting that *Astragalus* NSPs could highly improve pancreatic function by increasing β-cell mass and enhancing insulin effect [43]. Our findings further demonstrated the significant protective effect of NSPs on pancreatic tissue overall and specifically on β-cells. This was evident in the H&E staining images, where the increase of β-cell mass in treated mice was observed, attributed to the prevention of apoptosis due oxidative stress relief, and the restoration of their functionality through increased insulin synthesis, as previously noted in earlier sections. Similar histophathological observations were reported by Zhu et al. [44], showing that dietary polysaccharides from *G. atrum* enhanced β-cell mass, stimulated pancreatic islet expansion, and protected their structure from damage induced by a high-fat diet and STZ. Zhang et al. [45] reported comparable findings, demonstrating that mulberry leaf polysaccharides inhibited pancreatic islet cell apoptosis and improved insulin secretory capacity in STZ-induced diabetic rats.

The relationship between insulin resistance and obesity underscores the intricate interplay of metabolic factors that contribute to the pathogenesis of T2DM, highlighting the significance of addressing both insulin resistance and obesity in the management and prevention of this metabolic disorder [46]. The OGTT and ITT are also the main indicators of insulin resistance. Based on the data derived from the OGTT and ITT, it is evident that the administration of NSPs at a dose of 400 mg/kg effectively prevented the rise in blood glucose levels observed 30 min after glucose and insulin challenge. These findings strongly suggest that NSPs possess the capability to enhance peripheral glucose utilization and insulin sensitivity, indicating a notable hypoglycaemic effect. This aligns with the observed reduction in FBG starting from the second week of treatment, with this positive effect persisting throughout the entire experimental period. Similar findings were reported by Kumar et al. [14], showing that 4 weeks of treatment with gum polysaccharides led to a significant decrease in blood glucose levels during the OGTT and ITT, as well as a reduction in fasting FBG. Compelling evidence suggests that short-term increases in plasma free fatty acid (FFA) levels, commonly observed in individuals with both obesity and T2DM, lead to insulin resistance [47]. This correlation is commonly observed in tandem with the development of T2DM, which is often accompanied by dyslipidemia [48].

In the current study, diabetic mice showed a significant increase in TC, LDL-C, and TG, along with blood glucose elevation. However, NSPs treatment exhibited promising results in modulating serum lipid profile. Specifically, it effectively elevated HDL-C levels while concurrently reducing TC, TG, and LDL-C levels. The hypolipidemic effect of the extracted NSPs parallels that of rice bran polysaccharides, which primarily consist of xylose, rhamnose, mannose, galactose, arabinose, and glucose. These rice bran polysaccharides have been shown to regulate dyslipidemia in high-fat diet-induced mice by increasing HDL-C levels, decreasing LDL-C levels, and reducing liver fat deposition [49]. Additionally, polysaccharides from *Pleurotus ostreatus* have demonstrated similar effects in rats with hyperlipidemia induced by fat emulsion [50]. These findings highlight the potential therapeutic efficacy of NSPs in ameliorating dyslipidemia associated with diabetes.

Insulin resistance-induced lipolysis leads to heightened absorption of fatty acids by the liver, disrupting hepatic mitochondrial β-oxidation and promoting additional fat infiltration [51,52]. In rats fed a high-fat diet (HFD), dysregulated lipid metabolism has been linked to the activation of oxidative stress and inflammatory pathways in the liver, which contributes to the progression of non-alcoholic fatty liver disease (NAFLD) [53]. The results showed that higher doses of NSPs resulted in less fat accumulation in the livers of diabetic mice compared to the model group. Similar results were reported by [54], where the protective effect of *Ginkgo biloba* leaf polysaccharides has been approved on NAFLD mice by reducing lipid TG accumulation and lipid peroxidation. These findings were confirmed through H&E and Oil Red O staining analyses, clearly indicating a decrease in adipocyte size and diminished lipid accumulation. Liver steatosis is believed to significantly contribute to liver insulin resistance, leading to elevated gluconeogenesis and reduced glycogen synthesis, subsequently leading to disturbed glucose homeostasis [55]. Wang et al. [56] have studied the potential antidiabetic effect of *Rosa roxburghii Tratt* fruit polysaccharides, and noted an important increase in hepatic glycogen content after 8 weeks of treatment. Zhang et al. [45] observed a notable rise in liver glycogen levels in diabetic rats following treatment with mulberry leaf polysaccharides. In the present study, and after 4 weeks of NSPs administration, the low-dose treated mice expressed slightly higher liver glycogen levels, compared to the model group, suggesting NSPs as a potential glycogen synthesis enhancer or glycogen breakdown inhibitor. These findings indicate that NSPs support proper hepatocellular insulin action by sustaining hepatic glycogenolysis and promoting glycogenesis.

Elevated serum levels of AST and ALT are commonly associated with liver damage, serving as biological markers that connect liver disease with diabetes [57,58,59]. On the other hand, elevated levels of CRE and BUN are indicators of renal insufficiency, one of the most common complications of diabetes. This condition is associated with increased morbidity and mortality rates among diabetic patients. [60,61]. Polysaccharides from *Hizikia fusiforme*, mainly composed of fucose and mannose, at 400 mg/kg has demonstrated high efficacy in regulating blood AST and ALT and improved kidney dysfunction in diabetic mice [62]. The current findings revealed a positive correlation between elevated serum concentrations of AST, CRE, BUN, and the odds of diabetes. However, following a 4-week treatment with NSPs, the treated groups exhibited a significant decrease in AST and CRE levels compared to untreated mice. In addition, the analysis of blood insulin levels, a protein hormone released by islet β-cells, revealed that diabetic mice exhibited decreased blood insulin levels alongside elevated glucagon levels, suggesting impaired insulin synthesis by pancreatic β-cells, which suggests an imbalance in the regulation of blood glucose. NSPs-treated groups, on the other hand, showed higher blood insulin levels compared to the model group, along with a decreased level of glucagon and GLP-1, indicating a normal functioning of pancreatic β-cells by stimulating insulin secretion. Maintaining a balanced ratio of blood insulin and glucagon is crucial for the regulation of glucose metabolism, ensuring balanced gluconeogenesis and glycogenolysis, a state known as glucose homeostasis [63]. Additionally, glucagon action has also been linked to increased hepatic lipid β-oxidation and decreased *de novo* lipogenesis, which enhances fatty acid catabolism, resulting in reduced plasma triacylglycerol levels [64,65]. Similar findings have shown that polysaccharides extracted from *Enteromorpha prolifera* effectively lowered fasting blood glucose while enhancing the insulin sensitivity index in STZ-induced diabetic rats. Moreover, these polysaccharides significantly increased the number of islet β-cells and repaired pancreatic tissue damage in diabetic rats [66].

Persistent hyperglycemia and dyslipidemia induce the generation of ROS, leading to oxidative stress. This process is further exacerbated by proinflammatory cytokines released from adipose tissue and increased circulating FFA associated with obesity, which stimulate additional ROS production. Consequently, oxidative stress results in cellular and organ damage, contributing to the development of various complications associated with diabetes mellitus (DM) [67,68,69]. MDA, serving as an indicator of oxidative stress, exhibited elevated levels in diabetic mice according to our results. Conversely, treated mice demonstrated lower levels, indicating a potential mitigating effect of the treatment. The findings align with various studies, suggesting the efficacy of the anti-diabetic effect of dietary polysaccharides and their impact on managing oxidative stress [54,70,71]. Oxidative stress induced by hyperglycaemia and diabetes chronic dyslipidemia are thought to elevate the levels of pro-inflammatory cytokines [72]. Infiltrated macrophages release inflammatory cytokines, contributing to both local and systemic inflammation [73]. These findings are consistent with our results, demonstrating elevated levels of TNF-α, IL-6, and IL-1β in diabetic mice. Notably, these parameters showed a significant decrease following NSPs treatment. This suggests that NSPs have potential anti-inflammatory effects on diabetic mice. Liu et al. [74] demonstrated similar results, using polysaccharide extracted from *Pleurotus citrinipileatus,* that attenuated hepatotoxicity by increasing the IL-10 level and decreasing TNF-α and IL-6 levels. Comparable results were also reported by Wang et al. [12], using polysaccharide extracted from *Gynostemma pentaphyllum* herb.

## 5. Conclusions

This study underscores the promising impact of NSPs extracted from wheat beer, with xylose and mannose identified as predominant monosaccharides. Additional components, including galactose, glucose, and a trace of arabinose, contribute to the complex composition of these NSPs. Bioactivity analysis revealed a multifaceted positive influence, as NSPs exhibited inhibitory effects on α-amylase and pancreatic lipase, suggesting a potential to reduce blood glucose and lipid absorption by impeding enzyme activity. The binding capacity of NSPs to fat further indicated their potential as agents for lowering blood lipids. Physiologically, the NSPs extract demonstrated significant effects on various parameters. Notably, it lowered FBG levels. Furthermore, the extract markedly reduced serum lipid levels, including TC, TG, and LDL-C, while elevating HDL-C levels in treated groups, particularly at specific doses. The extract also demonstrated efficacy in mitigating liver lipid accumulation, contributing to potential preventative measures against fatty liver diseases. Kidney indexes exhibited improvements, and liver index markers, such as AST, were positively modulated, suggesting a protective effect on liver function. The study further highlighted the anti-oxidative properties of NSPs, as evidenced by a reduction in MDA levels, indicating attenuation of diabetes-induced oxidative stress. Concurrently, the NSPs extract exhibited anti-inflammatory effects by regulating the levels of TNF-α and IL-6. Histological analysis reinforced the protective effects observed, revealing improvements in pancreas structure and integrity, including enhanced β-cells activity. The liver sections displayed a notable decrease in fat deposition and inflammatory reactions, signifying a protective influence on liver tissue.

In conclusion, this comprehensive study suggests that NSPs, when administered at specific doses, positively influence blood sugar regulation, lipid profiles, and liver function in diabetic conditions. The multifaceted effects observed underscore the potential therapeutic benefits of NSPs extract, presenting avenues for further exploration in the management of diabetes and associated complications.

## Figures and Tables

**Table 1:** Inhibition rate of α-amylase and pancreatic lipase by NSPs.

Sample Concentration (mg/mL)	0.1	0.5	1.0	1.5	2.0	3.0
α-amylase inhibition rate (%)	11.36 ± 0.18	22.73 ± 0.22	27.27 ± 0.30	43.18 ± 0.27	70.45 ± 0.52	81.82 ± 0.85
Pancreatic lipase inhibition rate (%)	12.63 ± 0.11	17.79 ± 0.08	24.82 ± 0.27	27.04 ± 0.33	44.35 ± 1.05	45.73 ± 1.01

**Table 2:** Mice body weight and food intake. All data are shown as mean ± SD. Labeled means without a common letter differ significantly (*p* < 0.05).

	Initial Weight (g)	Final Weight (g)	The Amount of Weight Change (g)	Food Intake (g)
Control group	27.5667 ± 0.8595 ^bc^	29.5500 ± 0.6189 ^a^	1.9833 ± 0.5636 ^a^	271.6 ± 67.9 ^a^
Model group	26.6750 ± 0.8958 ^c^	31.1500 ± 2.6211 ^a^	4.4750 ± 2.5889 ^a^	302.35 ± 72.58 ^a^
Low-dose group	26.2000 ± 1.5621 ^c^	30.6000 ± 1.2288 ^a^	4.4000 ± 2.2517 ^a^	270.9 ± 65.72 ^a^
Medium-dose group	28.5000 ± 1.6279 ^ab^	30.7400 ± 2.0070 ^a^	2.2400 ± 2.3713 ^a^	291.9 ± 71.65 ^a^
High-dose group	29.3250 ± 0.4992 ^a^	32.0250 ± 1.6701 ^a^	2.7000 ± 1.7146 ^a^	274.1 ± 67.4 ^a^

## Data Availability

The original contributions presented in the study are included in the article, further inquiries can be directed to the corresponding author.

## Abbreviations

DM, Diabetes mellitus; FFA, Free fatty acids; HFD, High-fat diet; HSHF diet; High-sugar and high-fat diet; NSPs, Non-starch polysaccharides; NAFLD, Non-alcoholic fatty liver disease; T2DM, Type 2 diabetes mellitus.
